# Management of Juvenile Otosclerosis: A Systematic Review

**DOI:** 10.3390/children9111787

**Published:** 2022-11-21

**Authors:** Virginia Fancello, Luca Sacchetto, Chiara Bianchini, Andrea Ciorba, Daniele Monzani, Silvia Palma

**Affiliations:** 1 ENT & Audiology Unit, Department of Neurosciences, University Hospital of Ferrara, 44121 Ferrara, Italy; 2 Otolaryngology-Head and Neck Surgery Department, University Hospital of Verona, 37126 Verona, Italy; 3 Audiology, Primary Care Department, Ausl Modena, 41121 Modena, Italy

**Keywords:** juvenile otosclerosis, hearing loss, stapes surgery

## Abstract

Background. Otosclerosis can occur during childhood, resulting in the early onset of conductive hearing loss. The approach to a child with otosclerosis can present some difficulties in terms of diagnosis and treatment, and the literature on juvenile otosclerosis (JO) is still relatively limited. Aim. To explore the current approaches to JO, in order to clear the management of this condition and evaluate the outcomes and the possible complications of surgical treatment. Methods. A systematic review was performed according to PRISMA guidelines, searching Medline and Embase from January 2002 through to 30 September 2022. A total of 759 papers were identified but based on specified criteria, nine were included in this study. Results. There were 94 children affected by JO and treated by stapes surgery. According to the available data, Male: Female ratio was 1:3–4, whilst the mean ages ranged from 10 to 16.3 years at the time of stapes surgery. After stapes surgery, the target of ABG < 10 dB was achieved in most of the patients. Overall, the 4 complications were reported (4/94= 4%): stenosis of the external ear canal, deterioration of hearing, anacusis with vertigo, tinnitus. Conclusions. The heterogeneity of the available studies does not allow us to draw straight conclusions on this topic, currently. More data about the natural history of the disease in children could help in approaching the treatment correctly, and possibly in drawing guidelines. Studies with a prolonged follow-up could be helpful for assisting clinicians and families in taking the most favorable decision about treatment.

## 1. Introduction

Otosclerosis is a relatively common cause of hearing loss in adults, with a clinical prevalence estimated up to 0.3%. It is characterized by abnormal bone remodeling of the middle-inner ear, caused by bone resorption, new bone deposition, and vascular proliferation [1]. This process can occur independently or together at the stapes footplate, and/or at the cochlear capsule [2], even though otosclerotic focus begins at the fistula ante fenestram in 70% to 90% of cases. The location and extent of these lesions determine the symptomatology, which is most often due to conductive or mixed hearing loss. When the inner ear is affected, mixed or pure sensorineural hearing loss, tinnitus, and sometimes vertigo are present.

Even if considered an adult disease, it can appear during childhood, as well [3]. It is estimated that approximately 15% of patients with otosclerosis experienced hearing loss before the age of 18 [4].

A histologic study shows an incidence of less than 1% in children aged under 5 years (one case out of 161 temporal bones) and 4% in children aged between 5 and 18 years [5], indicating that clinical expression of the disease is even lower.

Etiology remains unclear, but genetic predisposition has been reported, and the pattern of inheritance is most often consistent with an autosomal dominant mutation with variable penetrance [6]. In 70–85% of cases, hearing loss is bilateral, developing initially in one ear before progressing to the other [7,8]. 

The main revealing feature is conductive hearing loss, and the differential diagnoses is with secretory otitis media, congenital stapes fixation, cholesteatoma, ossicular chain anomalies, and connective tissue disorders. Therefore, approaching a child with otosclerosis can present some difficulties in terms of diagnosis and treatment. Surgery, indications, and outcomes have been extensively examined in adults, but the literature for children is relatively limited.

In the past, surgery was postponed in most pediatric patients, and hearing aids were been prescribed. The first published series of pediatric patients successfully treated with stapes surgery dates back to 1980 [9], and various authors have reported positive surgical outcomes since then. However, the treatment of juvenile otosclerosis (JO) is still debated, and whether surgery should be performed regardless of age is still open to question. The risk of complications such as severe sensorineural hearing loss, anacusis, vertigo, and tinnitus is a compelling reason to postpone stapes surgery in pediatric patients, even though it is a regular practice in adults. 

The aim of this paper is to review the current approaches to JO in order to clear the management of this condition and evaluate the outcomes and possible complications of surgical treatment.

## 2. Materials and Methods

A literature search of English-language studies about otosclerosis in pediatric patients was performed using Medline and Embase databases to identify 20 years of relevant peer-reviewed papers. 

The keyword used for the search was “otosclerosis”. Additional filters applied were the age filter for children (0–18 years) and the language filter (English). A literature search was independently performed by two researchers (V.F., S.P.) who searched databases (January 2002–September 2022). The last search was performed on 30 September 2022. 

Inclusion criteria:

− subjects younger than 18 years old;
− definite description of hearing threshold;
− hearing loss related to JO;
− studies in English;

Exclusion criteria:

− case reports;
− studies containing duplicated data from other published work;
− studies published in languages other than English;
− studies without a description of hearing threshold;
− studies where the nature of stapes fixation was not specified.

The researchers have screened the selected literature according to inclusion and exclusion criteria. When titles and abstracts did not allow them, to identify one of the criteria mentioned, the full text of the study was reviewed to assess its relevance to this analysis. 

The extracted data included author name, publication year, sample size, interventions, outcome variables, number of reported patients, gender, pre and post-surgical hearing level, and follow-up time.

**Figure 1:**
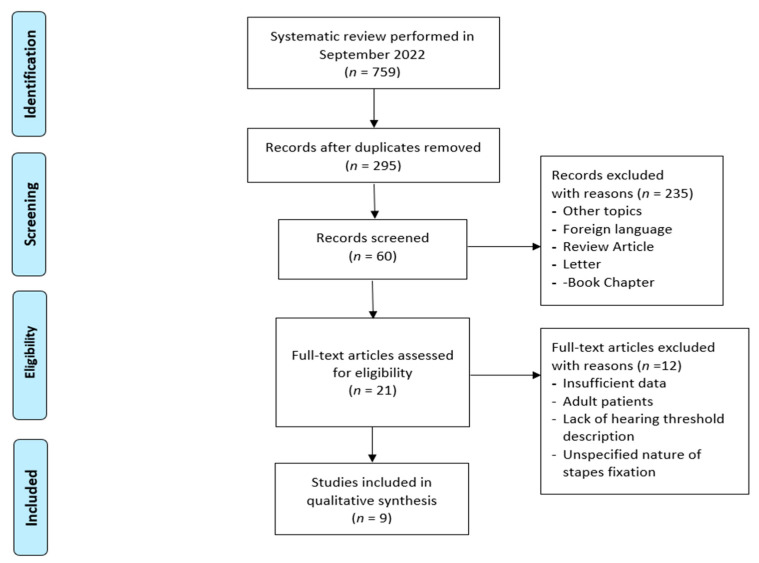
Literature evaluation and selection, according to PRISMA criteria (http://www.prisma-statement.org/ (last access 30 September 2022).

This review was conducted using the Preferred Reporting Items for Systematic Reviews and Meta-Analyses (PRISMA) guidelines. A total of 759 papers were identified in the search. The flow diagram is illustrated in Figure 1.

## 3. Results

Based on the previously specified criteria, nine papers were included in this review; all except one were retrospective. Two of the studies focused only on JO, whereas the others looked at congenital stapes fixation or other middle ear abnormalities (Table 1).

Data regarding those affected by JO were therefore extracted and further analyzed. All papers focused on surgical treatment.

The total number of children affected by JO and treated with stapes surgery was 94. Gender information was not supplied in four of the nine studies, based on the available data the Male: Female ratio was 1:3–4 (see also Table 2).

From seven studies, specific data on patients’ ages could be extracted. The patients’ mean ages ranged from 10 to 16.3 years at the time of stapes surgery. The youngest patient was 6.7 years old. 

The pure-tone average (PTA) air-bone gap (ABG) according to dB HL was calculated by averaging the patient’s thresholds for 0.5, 1, 2 and 3 or 4 kHz.

The values of ABG of those affected by JO, as reported by each paper, are summarized in Table 3. Hearing outcomes were calculated by comparison of preoperative and postoperative PTA ABG and were described in all papers. Overall, the target of ABG < 10 dB was achieved in most of the patients. The last follow up reported was >3 years in 4/9 cases. 

A preoperative computed tomography (CT) scan was used to rule out concomitant middle and/or inner ear malformations, examine the site of the otosclerotic process, and to evaluate the facial nerve. It was routinely performed in 8/9 series, while one of the older papers [17] described the use of Schuller views for the examination of the temporal bone.

Stapedotomy, with microscope assistance, was the most common surgical option performed. A perforator, micro drill, pick, or laser were the tools used to fenestrate the fixed footplate (Table 4). The complications reported were globally 4 (4/94= 4%): stenosis of the external ear canal which required revision surgery, deterioration of the hearing threshold, anacusis with vertigo, and tinnitus (see also Table 4).

## 4. Discussion and Conclusions

Otosclerosis is an early adult-onset disease, more prevalent in women than men, with a large minority of patients having a family history [6]. Diagnosis takes place during life, when clinical otosclerosis usually manifests itself as a conductive\mixed hearing loss caused by proceeding fixation of the stapes [1], and this review allowed us to confirm the prevalence of women, even in young patients, where fewer data among familiarity could be clearly extrapolated.

Hearing aids and surgical stapes replacement are the main therapeutic options proposed, and an accurate audiologic work-up is a crucial part of the diagnostic protocol of otosclerosis [19]. This is important not only to determine the air bone gap pre and post-operative, but also to differentiate JO from other causes of conductive hearing loss. Secretory otitis media is the main differential diagnosis with JO, also frequent in the juvenile population and even coexisting with otosclerosis, as reported by Markou [20]. Therefore, the precision in establishing air bone gap, based on masking techniques and immittance evaluations have a key role when considering a surgical approach to JO. Pure tone audiometry, tympanometry and acoustic reflexes must be combined into a test battery [21], especially in children as, the typical conductive hearing loss feature (a distinct notch-like decrease in bone conduction thresholds around 2000 Hz) is not always identified [22].

CT scans are a necessary step before surgery and, in recent years, high-resolution cone beam CT scans have been used to assess middle and inner ear with a significantly lower effective radiation dose than traditional CT scans [23]. The use of CT scan can identify otosclerotic foci and obliterative disease, and can be useful for surgical planning (i.e., for evaluating the risk of Gusher, or the presence of other anatomical variances).

A recent meta-analysis has evidenced that stapes surgery in children with JO or CSF appears to have an overall success rate (69.9%), described as an ABG reduction of 10 dB, but when JO diagnosis can be determined before surgery the postoperative rate rises to 80.2% [24]. Surgery, and in particular stapedotomy, is considered the treatment of choice to correct conductive hearing loss in this disease [25], but limited data were available on the long-term hearing effect, especially in children. This review has evidenced that most studies available are based on a follow up of around 3 years on average. 

The possible occurrence of complications is reason to postpone stapes surgery in pediatric patients, even though it is a regular practice in adults, as reported [26]. According to Yellon [26], the percentage of SNHL related to surgery is still relevant, and therefore, hearing aid evaluation should always be discussed and offered prior to surgery.

The youngest surgically treated child was six years old, but usually the mean age at surgery was ten years or higher. Postoperative care includes rest, avoidance of sneezing, and Valsalva maneuvers, therefore the patient’s compliance is crucial. For these reasons, involving children in the decision-making process is desirable.

Drawbacks: the significant heterogeneity of the studies, and subsequently the difficulties of the data processing, do not allow us to drive straightforward conclusions on this topic. Specifically, one of the main drawbacks was the identification of patients affected by JO, since the series often include findings from both congenital stapes fixation (CSF) and JO, which are similar conditions but with different surgical outcomes and long-term evolution.

In conclusion, the decision to treat surgically JO should be based on the features of the disease, children’s age, degree of hearing impairment, cochlear involvement, Eustachian tube function, children’s social and scholarly performance, and level of speech development. Since the recent literature among JO is limited, with the most available studies are retrospective, it is likely that more data about the natural history of the disease in children could help in correctly approaching the treatment, and possibly in drawing guidelines. Studies with a prolonged follow-up could be helpful for assisting clinicians and families in taking the most favorable decision about treatment. It is also desirable that further studies evaluating patient’s satisfaction and quality of life of those affected by JO become available in a near future. 

## Figures and Tables

**Table 1:** Ears treated in total by each Author, with respective intraoperative features. (Figure legend: Ref. = reference; CSF = Congenital stapes fixation; JO = Juvenile Otosclerosis; TS = Tympanosclerosis; SF = Stapes fixation).

Author	Ref.	Ears	Aetiology of Conductive Hearing Loss
Page JC et al.	[10]	67	49 CSF, 4 JO, 14 TS
Sobolewska A et al.	[11]	34	30 JO, 4 CSF with absence of the annulus
Vincent R et al.	[12]	41	41 JO
An YS et al.	[13]	28	22 CSF, 6 JO
Neilan ER et al.	[14]	24	17 CSF, 7 JO
Carlson ML et al.	[15]	44	27 CSF, 17 JO
Lescanne E et al.	[16]	9	9 JO
Bachor E et al.	[17]	12	5 OTS, 6 CSF, 1 anomaly of oval window
Welling DB et al.	[18]	46	15 TS, 4 JO, 21 CSF, 6 SF with middle and external ear malformations

**Table 2:** Demographics. Legend: ° = studies focused only on JO; * = not possible to extract this specific data on JO patients; F = female, M = male.

Author	Year	Country	Design of the Study	Time of the Study	# Patients	Mean Age (years)	Sex
Page JC et al.	2019	USA	Retrospective	2001–2017	4	11.5 +/− 3.4	2 F, 2 M
Sobolewska A et al.	2018	Spain	Retrospective	1987–2013	16	10	*
Vincent R et al. °	2016	France	Prospective	1991–2014	34	14.4 (range 8–18)	27 F,7 M
An YS et al.	2013	Korea	Retrospective	1999–2012	3	data on JO not extractable	2 F, 1 M
Neilan ER et al.	2013	USA	Retrospective	1999–2011	7	11.3 (range: 6.7–18)	*
Carlson ML et al.	2013	USA	Retrospective	1990–2011	14	16.3 +/− 2.3	*
Lescanne E et al. °	2007	France	Retrospective	1992–2005	7	13.7 (range 10–17)	6 F, 1 M
Bachor E et al.	2005	USA	Retrospective	1985–2000	5	11.6	*
Welling DB et al.	2003	USA	Retrospective	1994–2001	4	data on JO not extractable	*

**Table 3:** Hearing assessment and investigations. CT= Computed Tomography; BCHL = bilateral conductive hearing loss; ° = studies focused only on JO.

Authors	ABG Pre Op (Mean dB)	ABG Post Op (Mean)	Last Follow up	Imaging
Page JC et al.	38.3 (+/−7.6)	22.8 (+/−14.9)	>1 year post op Average 2.10 years (34.6 months) Not only JO cases	CT
Sobolewska A et al.	36.24	7.74	1 year post op	CT
Vincent R et al. °	25.7 (5.7)	3.0 (5.3) <10 dB in 93%, <20 dB in 98%	≥1 year post op Average 6.9 years (81 months)	
An YS et al.	32.8 (+/−8.4)	4.6 (+/−3.3)	>1 year post op Average 3.8+/1.9 years (46.8 +/− 23.5 months) Not only JO cases	CT
Neilan ER et al.	34.4 (+/−9.7)	7.2 (+/−5.4)	≥1 year post op Average 2.6 years	CT
Carlson ML et al.	27.5 +/− 5.9	8.8 (+/−7.9)	>1 year post op Average 3.7 years (43.1 months (range 12–112) Not only JO cases	CT
Lescanne E et al. °	mean closure 19 dB (+/−11.2)	6.5 dB (+/−3.7) mean closure 19 dB (+/−11.2)	>1 year post op Average 7.4 years (range: 2–15 years)	6/7 CT
Bachor E et al.	25.2	improved hearing postoperatively in 4 over 5 (16 dB)	1 year post op	2/12 had CT 10/12 Rx Schuller views
Welling DB et al.	41	12	>1 year post op Average 2.3 years (27.8 months) Not only JO cases	CT

° = studies focused only on JO.

**Table 4:** surgical technique, complication reported. * = not possible to extract this specific data on JO patients; # = case studies; ° = studies focused only on JO.

Authors	Surgical Technique	#	Complications
Page JC et al.	Microscope Transcanal approach Stapedotomy performed with -Fisch micro-perforator (3 mm) OR -Low-speed electric otologic drill with a 0.6 mm diamond bit	4	Not reported
Sobolewska A et al.	Microscope Transcanal approach (in one case a canaloplasty was performed simultaneously) Stapedotomy performed with -microdrill OR -CO_2_ laser	16	1 Stenosis of the external ear canal
Vincent R et al. °	Microscope Transcanal approach Stapedotomy performed with -KTP laser OR -CO_2_ laser Vein graft interposition in all cases	34	1 Postoperative sensorineural hearing loss (not anacusis)
An YS et al.	Microscope Transcanal approach Stapedotomy performed with -Skeeter drill	3	Revision surgeries were excluded from this Study
Neilan ER et al.	Microscope Transcanal approach Partial or total stapedectomy OR Stapedotomy performed with -laser	7	1 Acute vertigo and anacusis.
Carlson ML et al.	Microscope Transcanal approach OR Postauricular incision in case of limited exposure. Stapedectomy OR stapedotomy with micro drill in case of obliterated footplate	14	Not reported
Lescanne E et al. °	Microscope Transcanal approach Stapedectomy OR Stapedotomy with Laser	7	1 Tinnitus after surgery
Bachor E et al.	Microscope Transcanal approach stapedectomy OR stapedotomy (in 2 cases with CO2 Laser)	5	Not reported for JO cases
Welling DB et al.	Microscope Transcanal approach Stapedectomy OR Stapedotomy	4	* not possible to extract specific data for JO

° = studies focused only on JO.

## Data Availability

Not applicable.
